# Cardiometabolic Markers in Algerian Obese Subjects with and Without Type 2 Diabetes: Adipocytokine Imbalance as a Risk Factor

**DOI:** 10.3390/jcm14051770

**Published:** 2025-03-06

**Authors:** Hassiba Benbaibeche, Abdenour Bounihi, Hamza Saidi, Elhadj Ahmed Koceir, Naim Akhtar Khan

**Affiliations:** 1 Natural and Life Sciences Department, Biological Sciences Faculty, Algiers-1 University, Algiers 16000, Algeria; h.benbaibeche@univ-alger.dz; 2 Bioenergetics and Intermediary Metabolism Team, Laboratory of Biology and Organisms Physiology, University of Sciences and Technology Houari Boumediene, Algiers 16111, Algeria; abounihi@gmail.com (A.B.); saidi.hamza.dz@gmail.com (H.S.); e.koceir@gmail.com (E.A.K.); 3 Physiologie de la Nutrition & Toxicologie, UMR U1231 INSERM, Université Bourgogne Europe, 21000 Dijon, France

**Keywords:** obesity, inflammation, cytokines, adiposity, type 2 diabetes, cardiometabolic risk

## Abstract

**Background/Objectives**: An increase in body fat is linked to abnormalities in energy metabolism. We aimed at determining cardiometabolic risk in Algerian participants with obesity alone and with or without type 2 diabetes. The study measured the concentrations of circulating adipocytokines (leptin, adiponectin, resistin), tumor necrosis factor-α (TNF-α), and interleukin-6 (IL-6) to identify and examine how imbalances in adipocytokines may affect the parameters of cardiometabolic health. **Methods**: Algerian participants (*n* = 300) were recruited and divided into three groups: control, obese, and type 2 diabetics (with two sub-groups: with and without obesity). Insulin resistance was evaluated using HOMA-IR, while ELISA was used to measure adipocytokines. Atherogenic index in plasma (AIP), adiponectin-leptin ratio (ALR), and visceral adiposity index (VAI) were also assessed. One-way ANOVA was used to compare obesity and diabetes groups to the control one (*p* < 0.05). Logistic regression analysis was conducted to strengthen the robustness of statistical correlations. **Results**: Participants with reduced adiponectin-leptin ratio (ALR) and elevated levels of resistin, TNF-α, and IL-6 are found to be at higher risk of cardiovascular diseases. An imbalance in adipocytokine levels is caused by a decrease in adiponectin concentrations, and an increase in pro-inflammatory adipocytokines that maintain and exacerbate energy imbalance and induces hyperinsulinemia, exposing individuals to a high risk of cardiovascular diseases. **Conclusions**: Given that ALR is a functional biomarker of inflammation, insulin resistance, and adipose tissue dysfunction, targeting ALR could potentially be a therapeutic approach to coping with obesity-related cardiometabolic risks. Mediterranean diet, weight loss, and increased physical activity can be key components to promote healthy adipose tissue through the increase in ALR.

## 1. Introduction

Globally, there are more than a billion individuals who are obese [1]. The prevalence of obesity in Algeria is rapidly increasing, with 27.4% of individuals being affected by obesity [2]. Remarkably, two-thirds of obesity-related mortality is attributed to cardiovascular complications [3]. Body fat expansion, predominantly white adipose tissue (WAT), is observed in subjects with obesity. The WAT distribution includes fat from subcutaneous adipose tissue (SAT) and visceral adipose tissue (VAT). Excess VAT in obesity has been correlated with cardiovascular pathologies and type 2 diabetes, T2D [4]. According to the International Diabetes Federation, over 500 million individuals are suffering from diabetes. Recent estimations indicate that this number would rise to 783 million by the year 2045 [5]. In Algeria, the prevalence of diabetes continues to increase, reaching 14.4% in the population between the ages of 18 and 69 years [6]. Obesity results from several factors, like gender, genetic predisposition, high-calorie foods intake, less physical exercise, and dietary diversity [7,8]. Obesity is associated with dysregulation in circulating adipokines, i.e., high leptin and low adiponectin levels [9]. Leptin and adiponectin, mostly produced by adipocytes, are the main adipokines controlling eating behavior through their binding to arcuate nucleus [10] and lateral hypothalamus [11]. The adipokines regulate body fat homeostasis via modulation of insulin sensitivity [12], an increase in calorie expenditure [13] and a decrease in appetite through the inhibition of neuropeptide Y (NPY) expression [10]. Adiponectin reduces hepatic glucose production via gluconeogenesis inhibition, increases the flux of non-esterified fatty acids to the liver, stimulates glucose uptake in skeletal muscle, and increases energy expenditure through fatty acid oxidation [14]. In rodents, resistin has been shown to be released from adipocytes; however, in humans this hormone is secreted by macrophages within the adipose tissue [15]. Resistin is positively correlated with the production of proinflammatory cytokines, such as tumor necrosis factor-α (TNF-α), interleukin-6 (IL-6), and interleukin-1β, IL-1β [16].

The adiponectin–leptin ratio (ALR) has been recently reported to be a functional biomarker to assess dysfunction and inflammation of adipose tissue with an increased risk for cardiometabolic disease [17]. It has been demonstrated that ALR correlates more positively with IR, inflammation, and cardiometabolic risk factors than leptin or adiponectin [18]. Indeed, obesity is a major risk factor for the development of T2D through a multifactorial pathophysiology. In obesity, visceral fat accumulation is a key component of insulin resistance and dyslipidemia, leading to diabetes. The visceral adiposity index (VAI) is a predictor of diabetes, and the atherogenic index in plasma (AIP) is linked to atherosclerosis and cardiovascular diseases. Several studies have shown a strong link between obesity and cardiovascular diseases, and between type 2 diabetes and cardiovascular diseases.

A recent interesting review has proposed guidelines for the management of obesity with a focus on the diagnostic criteria for obesity and treatment options. Waist-to-height ratio (WHR) is a better predictor of metabolic risk and a gold standard measure of body fat distribution than BMI and WC [19]. In addition to classic risk factors, such as atherogenic lipoproteins and inflammation-related biomarkers, novel approaches in biomarkers include genetics, advanced imaging techniques, and artificial intelligence that have been used specifically in the identification of risk for developing atherosclerosis [20].

In this study, we have highlighted the link between the secretion of adipocytokines and cardiometabolic risk in obesity and type 2 diabetes. We expected that participants with obesity would exhibit an imbalance in their adipocytokine levels. We evaluated the cardiometabolic risk in Algerian adults by estimating cardiometabolic health parameters: AIP, ALR, and VAI. We determined the circulating concentrations of adiponectin, leptin, resistin, TNFα, and IL-6 in participants with obesity and type 2 diabetes with and without obesity, and compared them to those of the control group.

## 2. Materials and Methods

### 2.1. Study Design and Participants

This case-control study included 300 adults aged from 32 to 49 years, divided into three groups, with 100 participants per group. Control: 35 ± 2.79 years, having normal body mass index (BMI); and obese: 42.05 ± 1.17 years. The T2D group was divided into two subgroups; diabetes with obesity: 42.5 ± 2.3 years (*n* = 40) and diabetes without obesity: 44.32 ± 1.1 years (*n* = 60). The duration of diabetes varied from 2 to 4 years. Drug dosages were stable for at least 3 months. T2D participants did not exhibit comorbidities. They were treated with Metformin (850 mg twice daily). Throughout the study, no microbial infections were noticed. A fact sheet was prepared for the diagnosis of any complications in the recruited participants. The sex ratio was 0.94 (men/women). The exclusion criteria were type 1 diabetes, endocrinopathies, corticotherapy, or hormone therapy, renal, or any other complications. The clinical investigation was approved by the Algerian Ethics Committee of the Public Health Ministry (approval code: executive decree n° 10–90, dated 10 March 2010), and conformed to the Declaration of Helsinki. Informed consent was obtained from all the participants.

### 2.2. Assessment of Cardiometabolic Risk and Insulin Resistance

Insulin resistance was determined using the homeostasis model assessment insulin resistance (HOMA-IR) formula, calculated as follows: HOMA index = glucose (mmol/L) × insulin (mU/L)/22.5 [21]. Systolic (SBP) and diastolic (DBP) blood pressures were measured in participants after 5 min rest, by Omron 705 CP-type monitor. AIP was calculated according to the Nwagha formula [22]. The AIP was classified as low-risk (<0.11), moderate-risk (0.11–0.24), and high-risk > 0.24 [23].

### 2.3. Measurement of Anthropometric Criteria

Waist circumference (WC) was measured at the midpoint between the iliac crest and rib cage on the mid-axillary line. BMI was calculated by dividing the weight in kilograms by the square of height. The waist-to-hip ratio (WHR) was calculated to identify the nature of obesity, android, and gynoid. According to WHO, the WHR standards are set at 0.90 for men and 0.85 for women [24]. The percentage of body fat (BF %) was calculated for each participant according to the Deurenberg formula [25], as follows:

BF% = 1.2 × BMI + 0.23 × age − 10.8 × sex − 5.4 sex (men = 1, women = 0).

VAI was calculated using WC, BMI, triglycerides (TG) and HDL [26]. Sex specific formulae are:

women: VAI  =  WC/(36.58  +  1.89 × BMI) × (TG/0.81) × (1.52/HDL),

men: VAI  =  WC/(39.68  +  1.88 × BMI) × (TG/1.03) × (1.31/HDL).

### 2.4. Serum Samples Analysis

Fasting venous blood samples were collected. Serum glucose, TG, total cholesterol, low-density lipoprotein cholesterol (LDL), and high-density lipoprotein cholesterol (HDL) levels were quantified by enzymatic methods using an automatic biochemical analyzer (Cobas Integra 400^®^ analyzer, Roche Diagnostics, Meylan, France). HbA1c levels were determined by immunoturbidimetry using a Cobas Integra analyzer. Serum High-sensitive C-reactive protein (Hs-CRP) was quantified by the immunoturbidimetric method. Adiponectin, leptin, resistin, TNF-α, and IL-6 were determined in serum by ELISA (Millipore, Burlington, MA, USA). Serum insulin levels were determined using RIA (BioMerieux, Marcy-l’Étoile, France).

### 2.5. Statistical Analyses

Statistical analyses were performed by SPSS version 26. Data were calculated as the mean ± standard deviation (SD) with a significance level of *p* < 0.05. The Kolmogorov–Smirnov test was conducted to verify the normality. One-way ANOVA was used to compare obesity and diabetes groups to the control group, and the Tukey post hoc test was used. Power analysis indicated a minimum sample size of 159 for one-way ANOVA (G∗Power, 3.1.9.7), [27] with an effective size of 0.25, a significance level of 0.05, a power of 0.8, and three groups [28]. In order to achieve an adequate level of representation, the sample size was increased to 300 subjects. Pearson’s correlations were used to evaluate the association between VAI, ALR, and cardiometabolic biomarkers. Our data followed normal distribution. Logistic regression analysis was conducted to identify the association between obesity, type 2 diabetes-without obesity, and -with obesity and serum adipocytokine levels as well as ALR.

## 3. Results

### 3.1. Anthropometric Measurements

The sex ratio men/women was 0.94, expressing a high number of women (Table 1). The WC mean values showed an abdominal accumulation of adipose tissue in obese and diabetic groups versus control, which was more pronounced in diabetic participants with obesity. In the same way, BF% and the mean values of VAI and WHR were high in obese subjects in comparison with control participants. The WHR was in favor of an android obesity in obese, and diabetics with obesity vs. control. The mean values of WHR were above 0.85 for women and 0.90 for men, reflecting abdominal obesity as defined by WHO [24].

### 3.2. Metabolic Parameters

As shown in Table 2, participants with obesity, and diabetes were exposed to a high cardiometabolic risk, illustrated by insulin resistance (high insulin and HOMA-IR index values), glucose intolerance (a rise in HbA1c levels), and dyslipidemia. In participants with diabetes, glycaemia and HbA1C (>7%) levels were higher than control participants by 39%. Participants with obesity are normoglycemic but showed glucose intolerance. Obese and diabetic participants exhibited a high insulin-resistant state in comparison with control (*p* < 0.001); interestingly, diabetic subjects with obesity exhibited the highest value (Table 2). Participants with obesity and diabetes were characterized by hypertriglyceridemia with a decrease in HDL and an increase in LDL versus control group. All participants maintained a normotensive state. Table 2 shows that diabetic participants with obesity exhibited the highest value of Hs-CRP. AIP values favored a high risk of cardiovascular diseases in the obesity and diabetes groups. Indeed, AIP has been classified as high-risk at >0.24, the mean values in obese, diabetics with obesity, and diabetics without obesity were, respectively, 0.5, 0.48, and 0.33.

The Pearson correlations analyses showed a significant correlation between VAI and cardiometabolic biomarkers; WHR, TG, insulin, and AIP in participants with obesity, and diabetes with obesity (Table 3).

Interestingly, a significant inverse correlation exists between VAI and HDL. The strongest correlations were noticed with TG, AIP, and HDL. These results indicate a high risk of cardiometabolic diseases.

**Figure 1:**
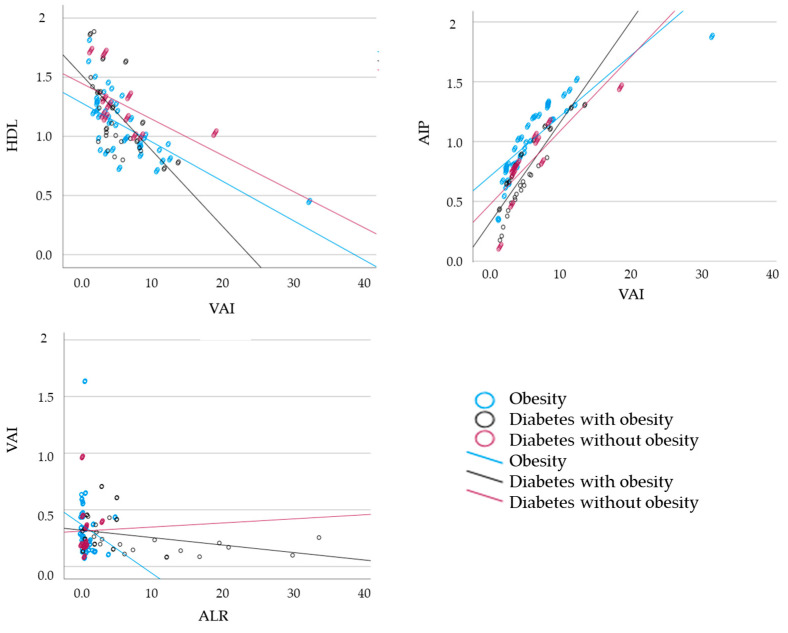
Correlation between HDL and VAI, AIP and VAI and between VAI and ALR.

For ALR, we observed a significant inverse correlation with cardiometabolic biomarkers (WHR, TG, insulin, AIP, and VAI) in the obesity and diabetes groups; however, ALR was positively correlated to HDL. These associations link low ALR-lipid risk factors with systemic inflammation that may lead to cardiometabolic diseases. We further employed 3 model of Logistic regression: Model 1, without adjustment; Model 2, adjustment by sex; and Model 3, adjustment by age (Table 4). Hence, obesity alone and type 2 diabetes with obesity were significantly associated with TNF-α, IL-6, leptin, adiponectin, resistin, and ALR even after adjusting for sex (Model 2) or age (Model 3). However, only IL-6, leptin, adiponectin, and resistin serum levels were found to be significantly associated with diabetes without obesity, even after adjusting for sex (Model 2) or age (Model 3). Figure 1 represents these key correlations.

### 3.3. Adipocytokine Levels

The TNF-α and IL-6 levels were high in participants with obesity, and diabetes with obesity, compared to control group (Table 5). The adipokines, particularly leptin and adiponectin, are sex dependent in both the groups (Table 5). Women of the control group exhibited higher serum levels of leptin (21%) and adiponectin (34%) than men of the control group, respectively, by 5.22 ± 0.40 ng/mL and 8.07 ± 1.15 µg/mL versus 3.25 ± 0.43 ng/mL and 6.04 ± 1.20 µg/mL (** *p* < 0.01).

Adiponectin levels were low in the obesity and diabetes groups compared to control participants (Table 5); the lowest level was noticed in participants with obesity. The ALR was significantly decreased in obesity and diabetes groups, especially in the group with obesity, where its value was highly reduced. Conversely to adiponectin, leptin concentrations were higher in participants with obesity, and diabetes, as compared to control group; diabetics with obesity showed the highest means values. Unlike with adiponectin and leptin, the resistin production was not sex-dependent. Resistin tended to increase in obesity, and diabetes.

## 4. Discussion

In Algeria, a Mediterranean diet is the traditional nutrition model. However, urbanization and nutritional transition have led to an imbalanced diet and deviated eating habits toward modern fast-food style and a high consumption of fat-rich and sweet food [29,30]. The Mediterranean diet could mitigate the harmful cardiovascular effects of being overweight and of obesity [31]. In fact, a recent study has shown that the Chrono Med Diet Score, a novel score for adherence to the Mediterranean diet, is inversely associated with visceral adiposity and cardiovascular risk [32]. The prevalence of obesity in Algeria is rapidly increasing, with 27.4% of individuals with obesity [2]. Moreover, the prevalence of diabetes continues to increase in Algeria, reaching 14.4% of the population between 18 and 69 years old [6]. In the current study, we investigated adipocytokine imbalance, cardiovascular risk, and systemic inflammation in Algerian participants suffering from obesity with or without type 2 diabetes.

Our study highlights the anti-inflammatory action of adiponectin and pro-inflammatory effects of leptin and resistin. In fact, the adiponectin receptor triggers various signaling pathways, including the activation of adenosine monophosphate-activated protein kinase (AMPK). This signaling pathway is associated with the recruitment of an adaptor protein, APPL1, which directly interacts with adiponectin receptors. Consequently, it leads to the phosphorylation of AMPK and MAPK, as well as to the activation of PPAR-α. These mechanisms collectively promote adipocyte differentiation, lipid metabolism, and glucose uptake [33]. Based on our results (see Table 5), adiponectin seems to act opposite to leptin and resistin in the control group and to reduce the secretion of proinflammatory cytokines (TNF-α and IL-6). In contrast to the control group, circulating leptin and resistin levels were increased along with serum proinflammatory cytokines in obesity and diabetes. Our findings showed that serum adiponectin levels were decreased in participants with obesity, and diabetes, with mean values under 5 μg/mL, in accordance with other populations with diabetes, obesity, and overweight [34,35]. The decrease in adiponectin levels can be explained by adipocyte autophagy [36]. Endoplasmic reticulum stress in adipocytes activates the autophagic degradation of adiponectin and reduces its levels in the blood. Studies on the Indian Pima population, which has the highest obesity and T2D prevalence, have shown that participants with elevated levels of serum adiponectin were less likely to develop T2D [37]. Interestingly, macrophages treated with adiponectin inhibit TNF-α and IL-1β secretion [38]. Hence, upregulation of TNF-α in 3T3 L1 adipocytes inhibits adiponectin and its downstream signaling [39]. The TNF-α level was higher in both the groups than the control group. In the obesity and diabetes groups, insulin resistance and hyperinsulinemia were observed despite high leptin levels. Leptin has been reported to block insulin secretion in pancreatic β-cells, whereas leptin resistance was found to provoke the continuous secretion of insulin, which may promote insulin resistance [40].

ALR is another marker of dysfunctional adipose tissue [18]. Negative correlations of ALR with WHR, VAI, insulin, and AIP reflect systemic inflammation. Similar correlations of ALR with body composition and cardiometabolic risk have been reported in childhood obesity and overweight, obesity, and diabetes in adults [41,42]. In the obesity and diabetes groups, the levels of pro-inflammatory cytokines such as resistin, leptin, IL-6, and TNF-α were high, which might have expanded the mass of dysfunctional adipose tissue. Hyperleptinemia has been demonstrated to amplify leptin proinflammatory effects and to promote TNF-α, IL-6, and IL-12 secretion and mRNA expression [43]. Likewise, a decrease in adiponectin has been reported to downregulate its anti-inflammatory effects. Indeed, adiponectin reduces NF-kB activation [44]. Resistin expression was increased in participants with obesity, supporting inflammation. Indeed, resistin might have upregulated IL-6, TNF-α, and its own expression through the NF-kB signaling pathway [45]. Obesity, adipocytokine imbalance, and inflammation are interconnected in a multifaceted, two-way relationship. Obesity can trigger alterations in adipocytokines and inflammatory responses, while inflammation can simultaneously contribute to the onset and progression of obesity. Chronic systemic inflammation may lead to insulin resistance, which impacts glycemia and fat storage, contributing to weight gain. Increased inflammatory markers lead to further fat accumulation and promote weight gain and inflammation. Moreover, in obese individuals, visceral adipose expansion leads to the release of high levels of leptin, resistin, TNF-α, and IL-6, and low levels of adiponectin, which promote inflammation and metabolic disorders. Despite hyperleptinemia in the obese, leptin resistance may appear due to leptin receptor dysfunction or the inhibition of leptin signaling pathways in hypothalamic neurons via the inhibition of JAK-STAT3 (Janus activated kinase-Signal transducer and activator of transcription 3) signaling pathways [46].

In the present study, the decreased concentrations of adiponectin and high levels of leptin in the obesity and diabetes groups seem to downregulate the cardiometabolic effects of the former. Cardiovascular risk was higher in the obesity and diabetes groups than in the control group. Adiponectin is a cardioprotective adipokine, while leptin and resistin can exacerbate cardiovascular dysfunction [47]. A previous study demonstrated that adiponectin, secreted from epicardial fat, can regulate cardiac function by increasing PPARγ signaling and protecting the heart against myocardial oxidative stress [48]. Similarly, serum leptin levels are associated with cardiovascular risk and metabolic syndrome, specifically in individuals with high leptin levels [49].

In the current study, the WHR values in the obesity and diabetes groups favored an increase in visceral adiposity, which is correlated with increased cardiovascular risk. The AIP values confirmed a high cardiometabolic risk in the obesity and diabetes groups; obtained values were greater than 0.24, as described earlier [26]. AIP has been described as a sign of cardiovascular disease [50]. The results of the current study show a positive correlation between VAI and cardiometabolic biomarkers, WHR, TG, AIP, and insulin, which underlies the high risk in accordance with other studies [51,52]. Furthermore, obese participants with high VAI are at risk of developing type 2 diabetes, as has been shown previously [53]. The mean values of VAI are high in obese and diabetic participants; the highest value is expressed in the group of diabetics with obesity. Several investigations have shown that VAI predicts the risk of diabetes in adults [54,55], and that it is correlated with the risk of vascular complications in diabetics [56,57]. A strong association was observed between ALR and HDL. However, ALR was inversely associated with TG levels. Similar findings have been previously reported [58]. The results of logistic regression analysis showed that obesity alone and type 2 diabetes with obesity were significantly associated with serum levels of TNF-α, IL-6, leptin, adiponectin, resistin, and ALR after adjusting for sex or age. However, only IL-6, leptin, adiponectin, and resistin serum levels were found to be significantly associated with diabetes without obesity, after adjusting for age. Additionally, the association between diabetes without obesity and serum IL-6 levels was weakened and lost significance after adjusting for age, which is probably due to the relatively younger age of our study population (Table 4). Consistent with this finding, previous studies have shown that a strong association exists between serum IL-6 levels and visceral fat mass only in older adults [59,60].

Adiponectin increases serum HDL and decreases serum TG via increased catabolism of TG-rich lipoproteins, which trigger both upregulation of HDL and reduction in TG by stimulating lipoprotein lipase activity [61]. Hypoadiponectinemia observed in obese and diabetic groups can be attributed to insulin resistance and dyslipidemia [62]. By interfering in insulin signaling pathways, adiponectin has been shown to inhibit hepatic glucose production (gluconeogenesis inhibition) and adipocyte lipolysis in VAT [63].

We would like to recall the concept of obesity paradox, which is based on the BMI value, suggesting that increased fat mass is less harmful and may be protective with increasing age, especially in older subjects with comorbidities [64,65]. However, a study of elderly people contradicts the concept of obesity paradox [66]. Hence, the term “BMI paradox” would be more appropriate, because BMI does not reflect body composition [64]. Moreover, in our study the participants were aged from 32 to 49 years and did not exhibit comorbidities. We also evaluated the WHR, known as the gold standard measure of body fat distribution and a predictor of cardiometabolic risk. We calculated the VAI, a mathematical model used to evaluate the distribution and function of fat. It is crucial to targeting the obesity that underlies cardiometabolic diseases. It may be difficult to change pathological state, but the challenge is to create long-term lifestyle changes that focus on prevention through adopting a healthy diet, as well as a Mediterranean diet, increased physical activity, and healthy eating behavior. Other factors, including lifestyle and genetic predisposition, may influence obesity-linked diabetes and cardiometabolic risks.

There are a few limitations to our study. We did not examine the physical activity, eating habits, and dietary patterns of participants. Since leptin is the main regulator of appetite and food intake, studying eating habits would be useful to deeply explain the adipocytokines imbalance and its link with cardiovascular risk. In addition, we included only obese participants and type 2 diabetic patients with and without obesity. It would have been interesting to involve more categories of BMI cutoff, such as overweight participants. The participants were from Algiers city; recruitments of participants from others cities in Algeria would also be better for future research. Moreover, our findings need to be confirmed in a larger population in order to better understand the role of adipocytokines in cardiometabolic health, and their possible use in clinical practice in the near future.

## 5. Conclusions

We conducted the study on an Algerian population as a contribution to the literature of previously published work using cardiometabolic markers in other populations. The current study provides, for the first time, worthy data on the involvement of adipocytokines imbalance as a cardiometabolic risk in obesity and obesity-related type 2 diabetes, and their links with cardiometabolic health biomarkers. Targeting ALR can be a therapeutic way to prevent and treat obesity related cardiometabolic risk. In this way, a Mediterranean diet, weight loss, and increased physical activity can be key components to increasing ALR and promoting healthy adipose tissue.

## Figures and Tables

**Table 1:** Anthropometric Profile.

P/Groups	Control (n = 100)	Obesity (n = 100)	Diabetes (n = 100)	
			With Obesity (n = 40)	Without Obesity (n = 60)
BMI (kg/m^2^)	22 (3) ^W^	34 (2) ^W^ ***	33.7 (2.96) ^W^ ***	24.3 (3.2) ^W^ *
	21 (2) ^M^	32 (2) ^M^ **	33.6 (1.9) ^M^ **	26.9 (1.8) ^M^ *
WC (cm)	77.3 (2.2) ^W^	107 (7.1) ^W^ ***	109 (9.8) ^W^ ***	87.7 (9.6) ^W^ **
	81.3 (5.1) ^M^	110 (3.2) ^M^ ***	112 (4.9) ^M^ ***	99.1 (6.1) ^M^ ***
WHR	0.83 (0.03) ^W^	1.03 (0.02) ^W^ ***	1.02 (0.05) ^W^ **	0.93 (0.01) ^W^ ***
	0.86 (0.01) ^M^	1.09 (0.01) ^M^ ***	1.04 (0.06) ^M^ ***	0.97 (0.01) ^M^ ***
BF (%)	6.5 (0.9) ^W^	26.2 (3.5) ^W^ ***	27 (2.4) ^W^ ***	17.2 (1.3) ^W^ ***
	2.2 (0.6) ^M^	18.7 (2.3) ^M^ ***	26.2 (3.5) ^M^ ***	21.2 (2.6) ^M^ ***
VAI	3.7 (2.02)	6.02 (4.7) **	7.1 (1.3) **	6.32 (3.9) **

P: parameters; ^M^: men; ^W^: women; BMI: Body mass index. WC: Waist Circumference. WHR: Waist-to-hip Ratio; BF %: Body fat percentage. VAI: Visceral adiposity index. * *p* < 0.05; **: *p* < 0.01; ***: *p* < 0.001.

**Table 2:** Metabolic Parameters.

P/Groups	Control (n = 100)	Obesity (n = 100)	Diabetes (n = 100)	
			With Obesity (n = 40)	Without Obesity (n = 60)
Glycaemia (mmol/L)	4.6 (1.5)	5.3 (1.6)	7.5 (0.5) **	7.2 (0.4) **
Insulinemia (pmol/L)	8.8 (1.7)	42.8 (4.7) ***	46.1 (8.2)	43.5 (5.9)
HOMA	1.9(0.07)	10.4 (1.3) ***	15.83 (3.86) ***	11.32 (1.3) ***
HbA1C (%)	4.5 (1.5)	5.1 (2.6)	6.5 (1.2)	5.4 (1.1)
Triglycerides (mmol/L)	1.6 (0.2)	3.7 (0.5) ***	3.9 (0.4) ***	3.2 (0.2) ***
Cholesterol (mmol/L)	3.9 (0.18)	4.9 (0.8) *	4.8 (0.7) ***	4.4 (0.8) *
HDL (mmol/L)	1.5 (0.08) ^W^	1.23 (0.04) ^W^ *	1.2(0.12) ^W^ *	1.4 (0.15) ^W^ *
	1.2 (0.04) ^M^	0.92 (0.04) ^M^ **	1.2(0.07) ^M^ *	1 (0.1) ^M^ *
LDL (mmol/L)	2.4 (0.54)	3 (0.8) *	3.1 (0.3) *	2.8 (0.22) *
Hs-CRP (mg/L)	3.5 (1.2)	5.6 (0.1) **	6.4 (0.3) **	4.8 (0.2) **
SBP (mm Hg)	121 (12)	137 (3) *	138 (4) **	135 (3) **
DBP (mm Hg)	73 (5)	82 (5)	84 (2) **	83 (3) **
AIP	0.1	0.5 ***	0.48 **	0.33 **

P: parameters; ^M^: men; ^W^: women; HOMA: Homeostasis Model Assessment; HDL: high density lipoprotein; LDL: low density lipoprotein. SBP: systolic blood pressure; DBP: diastolic blood pressure. Hs-CRP: High sensitive C reactive Protein. AIP: plasma atherogenic index. *: *p* < 0.05; **: *p* < 0.01; ***: *p* < 0.001.

**Table 3:** Pearson correlations between ALR, VAI and measures of cardiometabolic health.

Groups	Obesity (n = 100)	Diabetes (n = 100)	
		With Obesity (n = 40)	Without Obesity (n = 60)
VAI			
WHR	0.2 *	0.2 *	0.4 *
TG	0.9 ***	0.9 ***	0.5 **
HDL	−0.6 ***	−0.6	−0.8 ***
Insulin	0.5 *	0.2 *	0.2 *
AIP	0.8 ***	0.81 *	0.9 ***
ALR			
WHR	−0.3 *	−0.12	−0.2 *
TG	−0.1	−0.3 *	−0.4 *
HDL	0.1	0.3 *	0.2 *
Insulin	−0.2 *	−0.3 *	−0.3 *
AIP	−0.1	−0.2 *	−0.5 *
VAI	−0.2 *	−0.3 *	−0.4 *

VAI: visceral adiposity index; WHR: waist-to-hip ratio; TG: triglycerides; HDL: high density lipoprotein; AIP: atherogenic index of plasma; ALR: adiponectin-leptin ratio. * *p* < 0.05, ** *p* < 0.01, *** *p* < 0.001.

**Table 4:** Association between simple obesity, type 2 diabetes: without obesity and with obesity and serum adipocytokine levels as well as ALR.

P/Groups	Control (n = 100)	Obesity (n = 100)	Diabetes (n = 100)	
			With Obesity (n = 40)	Without Obesity (n = 60)
TNF-α (pg/mL)				
Model 1				
OR (95%CI) *p*	1	1.267 (0.850; 1.887) 0.04	1.298 (0.871; 1.935) 0.01	1.234 (0.833; 1.828) 1.234
Model 2				
OR (95%CI) *p*	1	1.249 (0.846; 1.844) 0.04	1.273 (0.862; 1.881) 0.02	1.226 (0.835; 1.802) 0.298
Model 3				
OR (95%CI) *p*	1	1.341 (0.890; 2.021) 0.01	1.376 (0.912; 2.075) 0.02	1.310 (0.873; 1.966) 0.192
IL-6 (pg/mL)				
Model 1				
OR (95%CI) *p*	1	1.236 (1.000; 1.527) 0.04	1.209 (0.974; 1.501) 0.04	1.120 (0.918; 1.367) 0.04
Model 2				
OR (95%CI) *p*	1	1.254 (1.005; 1.564) 0.045	1.236 (0.986; 1.551) 0.03	1.110 (0.901; 1.367) 0.02
Model 3				
OR (95%CI) *p*	1	1.259 (0.998; 1.589) 0.032	1.233 (0.973; 1.563) 0.043	1.145 (0.917; 1.430) 0.231
Leptin (ng/mL)				
Model 1				
OR (95%CI) *p*	1	1.279 (1.111; 1.472) ˂0.001	1.282 (1.112; 1.478) ˂0.001	1.169 (1.018; 1.342) 0.027
Model 2				
OR (95%CI) *p*	1	1.466 (1.201; 1.788) ˂0.001	1.440 (1.178; 1.759) ˂0.001	1.298 (1.069; 1.576) 0.008
Model 3				
OR (95%CI) *p*	1	1.602 (1.232; 2.082) ˂0.001	1.571 (1.207; 2.044) ˂0.001	1.415 (1.092; 1.834) 0.009
Adiponectin (µg/mL)				
Model 1				
OR (95%CI) *p*	1	0.638 (0.507; 0.803) ˂0.001	0.751 (0.593; 0.951) 0.017	0.809 (0.697; 0.940) 0.006
Model 2				
OR (95%CI) *p*	1	0.619 (0.484; 0.791) ˂0.001	0.707 (0.541; 0.923) 0.011	0.813 (0.699; 0.944) 0.007
Model 3				
OR (95%CI) *p*	1	0.622 (0.489; 0.791) ˂0.001	0.712 (0.553; 0.916) 0.008	0.754 (0.630; 0.903) 0.002
Resistin (ng/mL)				
Model 1				
OR (95%CI) *p*	1	4.856 (1.738; 13.569) 0.003	3.879 (1.371; 10.974) 0.011	3.592 (1.323; 9.757) 0.012
Model 2				
OR (95%CI) *p*	1	5.123 (1.822; 14.406) 0.002	4.036 (1.420; 11.472) 0.009	3.609 (1.326; 9.820) 0.012
Model 3				
OR (95%CI) *p*	1	17.602 (2.395; 129.392) 0.005	14.178 (1.916; 104.893) 0.009	13.216 (1.824; 95.785) 0.011
ALR				
Model 1				
OR (95%CI) *p*	1	0.013 (0.002; 0.090) ˂0.001	0.044 (0.006; 0.323) 0.002	0.831 (0.621; 1.113) 0.215
Model 2				
OR (95%CI) *p*	1	0.007 (0.001; 0.058) ˂0.001	0.043 (0.005; 0.345) 0.003	0.784 (0.570; 1.079) 0.136
Model 3				
OR (95%CI) *p*	1	0.006 (0.001; 0.048) ˂0.001	0.035 (0.004; 0.290) 0.002	2.237 (0.626; 7.993) 0.215

**Table 5:** Adipocytokine Levels.

P/Groups	Control (n = 100)	Obesity (n = 100)	Diabetes (n = 100)	
			With Obesity (n = 40)	Without Obesity (n = 60)
TNF-α (pg/mL)	6 (1.8)	8.4 (0.7) **	9.8 (3) **	6.3 (2.2) *
IL-6 (pg/mL)	7.6 (1.4)	12.8 (1.9) ***	10.3 (1.4) **	9.1(1.3) **
Leptin (ng/mL)	5.5 (1.9) ^W^	31.3 (3.3) ^W^ ***	41.4 (7) ^W^ ***	12.4 (3) ^W^ ***
	4.6 (1.2) ^M^	13.1 (3.2) ^M^ ***	20 (9.1) ^W^ ***	5.7 (1.1) ^M^ *
Adiponectin (µg/mL)	8 (1.1) ^W^	3.8 (0.4) ^W^ **	5.1 (0.2) ^W^ **	5.8 (1.4) ^M^ *
	6 (1.2) ^M^	1.8 (0.3) ^M^ ***	3.2 (0.8) ^M^ ***	4.1 (0.6) ^M^ *
Resistin (ng/mL)	3.8 (0.6) ^W^	6.1 (0.9) ^W^ *	5.5 (0.7) ^W^ **	4.1 (0.4) ^W^ **
	3.2 (0.8) ^M^	6.8 (0.7) ^M^ ***	6.7 (1.4) ^M^ **	5.01 (0.3) ^M^ **
ALR	1.1 (0.6) ^W^	0.1 (0.02) ^W^ ***	0.07 (0.02) ^W^ ***	0.5 (0.3) ^W^ **
	1.8 (0.6) ^M^	0.2 (0.08) ^M^ ***	0.23 (0.1) ^M^ ***	1 (0.1) ^M^ *

P: parameters; ^M^: men; ^W^: women; TNF-α: Tumor necrosis factor alpha; IL-6: Interleukin-6; ALR: Adiponectin-leptin ratio. *: *p* < 0.05; **: *p* < 0.01; ***: *p* < 0.001.

## Data Availability

The datasets generated and analyzed during the current study are not publicly available, as individual privacy could be compromised.
